# Flow Cytometry, a Unique Biotechnology in Medical Applications

**DOI:** 10.3390/jcm11206198

**Published:** 2022-10-20

**Authors:** Claude Lambert

**Affiliations:** 1 Laboratoire D’immunologie Clinique, Hôpital Nord, 42000 Saint-Étienne, France; claude.lambert@chu-st-etienne.fr; 2 LCOMS/ENOSIS Environmental Toxicology, University of Lorraine, 57000 Metz, France

It is good to see that the *Journal of Clinical Medicine* has recently published several good papers, highlighting the special input of cell analysis using flow cytometry. These papers use flow cytometry in a very wide field, from very small cell-derived particles to huge cells, such as myocytes, and different detection technologies from the conventional fluorescence to mass spectrometry. This reflects the quality of the papers correctly using methods of high potential.

Flow cytometry (FCM) is a unique biotechnology in the sense that it gives very precise information on each individual out of a complex mixture of particles. FCM has recently been invented and is in permanent development. FCM’s precision is due to its unique capability of simultaneously measuring each individual event, with more and more parameters, routinely six to eight but sometimes even more [1]. FCM does not provide a global view or a “standard individual type” of the particle subpopulations with mean values and standard deviation, but it gives information on each individual particle. Cells can then be qualified according to the quantitative expression of markers and can be separated into two or more subclasses with each parameter analyzed. The more parameters you measure, the better you can classify the populations. As an example, using 8 parameters, more than 256 homogeneous groups (clusters) can be determined and 10 parameters can identify more than 1024 clusters. FCM analyzes at high speed, meaning 200 up to 1000, or even more, cells per seconds. Thus, acquiring several hundred thousands of cells makes it possible to have enough representatives for each of the numerous groups. Very rare subsets with homogenous phenotypes can be identified. Recent mathematical tools have been developed to help in clustering cell sub populations, in a non-supervised way, taking into consideration each quantitative parameter on each cell.

In FCM, particles are analyzed in a stream, of less than 200 µm, passing if front of laser beams of approximately 10 µm height, 100 µL wide. Cytometer easily have three or more laser beams in parallel. Cells in the flow need to be perfectly aligned with each laser beam. Thus, FCM is perfectly adapted for analysis of particles between 1 and 20–100 µm specially. Cells in suspension in biological fluids are particularly adapted for FCM analyzes. However, there are methods to make cells in tissue or adhering on culture plate to be resuspended for the FCM analysis with the cost of some cell damages, residual aggregates, and frequently a lot of debris making electronic noise.

The unique fact that information is given for each individual cell brings new approaches in biological analysis. “Statistics”, here, have all their meaning, trying to give a “general idea” on the population, considering it has a “normal” Gaussian distribution. In brief, in an ideal world, any parameter from most populations has some symmetrical variability around the its mean value. However, classical statistics tend to neglect the minorities and outliers. It is particularly obvious that counting the main large subpopulations (as an example T CD4+/T CD8+ T cells) do not necessarily give helpful information on the specific immune function that is protecting the body against one pathogen or eventually causing trouble by mismatching with a constituent of the body itself.

Furthermore, cell diversity in biological fluid is not limited to its lineage that can be easily identified and counted separately, such as B cells, CD4 + T cells, monocytes, or basophils, etc. However, inside each lineage, each individual cell has its own age, maturation status, capacity for migrating to different tissues (homing as an example, to lymph node, bone marrow, peripheral tissues), and eventual activation or exhaustion status according to its individual experience of life. The cell variability is evidenced even in experimental culture from a monoclonal cell line, where each individual cell has particularities regarding its resting/activity status, its proliferation rate and cell cycle step, or its metabolic condition.

Giving a clear description of a “typical phenotype” makes results very clear but is in fact only partially revealing of the reality of the population diversity, neglecting individual cell lifetime, forgetting some minorities, and even more frequently the rare outlier individuals. Additionally, sometime, it is the rare outlier that causes trouble. This is particularly the case in infections, for example, where the specific T cells are able to specifically fight the intruder, representing only a very small minority out of the total T cell population covering the whole repertoire. This is also true for tumor cells. Chemotherapy can treat the vast majority of tumor cells. However, few outliers may manage to escape the treatment toxicity. Rare few escapes can then expand exponentially, taking advantage of the empty space led by massive cell destruction and progressively become the leading clone.

The precision of FCM analysis also makes possible the collection of most information out of a single tube. This is of high interest in sparing time for sample analysis. It also helps extract the most information out of precious samples that contain very small concentrations of cells, such as cerebrospinal fluid (CSF) or fine needle aspiration (FNA) of tissue or tumor. As an example, Carla Griesel and colleagues [2] have shown the performance of FCM in identifying B and T cells as chronic proliferative B cells or acute leukemic phenotypes, in very high agreement with pathological analysis but with higher precision.

The high precision of analysis out of very large populations gives the possibility to identify very rare events of interest, such as circulating tumoral cells or monitoring the decline of leukemia cells under treatment (minimal residual disease), giving a more precise idea on the treatment efficacy. Caroline Dix and colleagues [3] addressed the different strategies in declining monitoring (tumor) cell concentration in peripheral blood and to what extent FCM can help in decision making whenever the disease is over or if there is still the need to complete or intensify high-dose chemotherapy. FCM sensitivity can detect as low as 1 tumor cell out of 10^6^ leukocytes, as compared to 1 out of 20 by standard morphology, assuming the tumor cell has a Leukemia-associated immunophenotype (LAIP) that can be identified by immunodetection. One must keep in mind that this LAIP may change over time due to malignant cell instability.

FCM detection is largely based on the use of fluorescence that is very sensitive and is available in many different colors. This makes it possible to simultaneously measure multiple labelings that can be quantified separately using filters on the optical benches and computing tools. With this technology, it is possible to do more than 10, potentially up to 20 multiple immunolabelings. This will probably be the limitation of the method. However, with new innovations, it is now possible to go further, taking pictures of each cell (image cytometry), separating more fluorochromes by taking into consideration their full spectrum (spectral cytometry) or by replacing fluorescence and photodetectors by metals and mass spectrometry. Using mass cytometry, Lucia Lisa Petrilli and colleagues [4] could simultaneously analyze 16 antigens of embryonal rhabdomyosarcoma (eRMS) in an experimental model. Using a mathematical approach, they could clearly find the main tumor constituents, such as pericytes and also four minor unexpected clusters, classified a “other cell types” that could not be identified using the classical strategy. This open perspective exploring the role of these new cell clusters that need to be further characterized.

Analyzing cells into different conditions in consecutive tubes makes it possible to do functional tests on cells. As an example, Ana Reula and colleagues [5] measured the capacity of nasal epithelial cells in producing oxygen radicles. Oxidation makes some molecules become fluorescent. Fluorescence is then directly proportional to the production of free radicles produced in individual cells, depending on the stimulation, as well as the cell proper capacity. More functional tests have been used and the procedures can be very simple and rapid, stimulating cells just before or even during the incubation time for immunolabelling [6].

Small cells, such as platelets, can also be analyzed by FCM, although they are fragile and very sensitive to pre-analytical conditions. Alexandre Mansour and colleagues [7] explored platelet stress induced by the contact with plastic tubes during extracorporeal circulation. This ex vivo activation affects the thrombotic capacity of platelets and increases the risk for venous thromboembolism after re-injection to the patient. What apply to extracorporeal membrane oxygenation probably also acts during other extracorporeal circulation, such as in hemodialysis, plasma exchanges, or even plasma or platelets donation.

Going to an even smaller range, below 1 mm, very close to the limit of detection, it is possible to detect extracellular vesicles (EV). This, of course, requires very meticulous precautions. EV are tiny fragments released by cells, especially under activation and can diffuse widely in blood, specially from cells are enclosed in some compartments. They are composed of cell membrane and cytoplasm, thus, reflecting the identity of their mother cell, such as platelets (CD41, CD36+, or CD61), endothelial cells (CD31+, CD105+), leukocytes (CD45+), B-lymphocytes (CD19+) and T-cells (CD3+), monocytes (CD14+), or erythrocytes (CD235a+). The adhesion capacity of EV can also be measured with expression of Integrin alphaV (CD51), ICAM-1 (CD54), endothelial E-selectin (CD62E), P-selectin (CD62P), or PECAM-1 (CD31). EV are not only tiny messages from cells, especially from very active cells, they may also have some regulating functions due to their membrane expression of ligands. EV also carry cytoplasmic component with eventual transmissible activity, such as micro-RNAs regulators. As an example, neurons in the central nervous system are sequestered by the blood–brain barrier that only EV can cross. Thus, Jakub Soukup [8] used the opportunity to get information on multiple sclerosis (MS) neuronal inflammation by analyzing EV of neural origin in peripheral blood. Even more, Hee Cheol Yang [9] managed to combine to advanced technology, making possible the EV characterization together with intravesicular micro RNA detection using fluorescent molecular beacons.

Macromolecular complexes can also be analyzed by flow cytometry. Emmanuel Schneck and colleagues [10] analyzed neutrophil extracellular traps (NETs), a sort of molecular package produced by granulocytes that contain a mixture of proteins entrapped in a DNA wool ball. NETs trap pathogens and closely interact with platelets favoring thrombosis. Emmanuel Schneck has shown that NETs can be detected in plasma by FCM and their content brings information on granulocytes activity in some diseases, such as in septic shock, as major players in immunothrombosis.

In conclusion, flow cytometry has exceptional performances to analyze cells or cell satellites with exceptionally high precision, in their phenotypes diversity not excluding rare outliers, as well as in their functional status. This gives the chance to approach biology not only looking at it as schematic standard types of cell subsets but as heterogeneous, highly diverse populations. Mathematical tools are available to change our approach and consider biology in its complexity.
